# The Breast Cancer Protooncogenes HER2, BRCA1 and BRCA2 and Their Regulation by the iNOS/NOS2 Axis

**DOI:** 10.3390/antiox11061195

**Published:** 2022-06-17

**Authors:** Katie Lin, Stavroula Baritaki, Silvia Vivarelli, Luca Falzone, Aurora Scalisi, Massimo Libra, Benjamin Bonavida

**Affiliations:** 1 Jonsson Comprehensive Cancer Center, Department of Microbiology, Immunology and Molecular Genetics, David Geffen School of Medicine, University of California, Los Angeles, CA 90095, USA; katielin@mednet.ucla.edu; 2 Laboratory of Experimental Oncology, Division of Surgery, School of Medicine, University of Crete, 71003 Heraklion, Crete, Greece; baritaks@uoc.gr; 3 Department of Biomedical and Biotechnological Sciences, University of Catania, 95030 Catania, Italy; silvia.vivarelli@unime.it (S.V.); m.libra@unict.it (M.L.); 4 Occupational Medicine Section, Department of Biomedical and Dental Sciences and Morphofunctional Imaging, University of Messina, 98123 Messina, Italy; 5 Epidemiology and Biostatistics Unit, IRCCS Istituto Nazionale Tumori “Fondazione G. Pascale”, 80131 Naples, Italy; l.falzone@istitutotumori.na.it; 6 Italian League against Cancer, 95030 Catania, Italy; a.scalizi@unict.it; 7 Research Centre for Prevention, Diagnosis and Treatment of Cancer, University of Catania, 95030 Catania, Italy

**Keywords:** breast cancer, iNOS/NOS2, nitric oxide, HER2, BRCA1, BRCA2, TNBC

## Abstract

The expression of inducible nitric oxide synthase (iNOS; NOS2) and derived NO in various cancers was reported to exert pro- and anti-tumorigenic effects depending on the levels of expression and the tumor types. In humans, the breast cancer level of iNOS was reported to be overexpressed, to exhibit pro-tumorigenic activities, and to be of prognostic significance. Likewise, the expression of the oncogenes HER2, BRCA1, and BRCA2 has been associated with malignancy. The interrelationship between the expression of these protooncogenes and oncogenes and the expression of iNOS is not clear. We have hypothesized that there exist cross-talk signaling pathways between the breast cancer protooncogenes, the iNOS axis, and iNOS-mediated NO mutations of these protooncogenes into oncogenes. We review the molecular regulation of the expression of the protooncogenes in breast cancer and their interrelationships with iNOS expression and activities. In addition, we discuss the roles of iNOS, HER2, BRCA1/2, and NO metabolism in the pathophysiology of cancer stem cells. Bioinformatic analyses have been performed and have found suggested molecular alterations responsible for breast cancer aggressiveness. These include the association of BRCA1/2 mutations and HER2 amplifications with the dysregulation of the NOS pathway. We propose that future studies should be undertaken to investigate the regulatory mechanisms underlying the expression of iNOS and various breast cancer oncogenes, with the aim of identifying new therapeutic targets for the treatment of breast cancers that are refractory to current treatments.

## 1. Introduction

Several reports have described the role of inducible nitric oxide synthase (iNOS) in various cancers and its clinical significance [1,2,3,4,5,6]. Such reports have indicated that the expression of iNOS and nitric oxide (NO) in some cancers is anti-tumorigenic. For example, it was found that in mice, iNOS is anti-tumorigenic in colon cancer cells [7,8]. Additionally, when iNOS was transfected into murine melanoma cells, iNOS inhibited tumorigenesis and metastasis [9,10]. However, other reports demonstrated the pro-tumorigenic activity of NO. One study found that iNOS is induced both in the epithelial cells and the environmental stroma community of a tumor, which promotes tumorigenesis [3,11]. Findings in breast cancer also reported that iNOS expression was correlated with tumor progression and was of prognostic significance [12,13,14,15,16].

Various mechanisms have been postulated for the contrasting role of iNOS/NO in cancers. Specifically, it was found that the dual role of iNOS is dependent on its concentration, cell type, and environment [3]. High concentrations of iNOS-induced NO were found in human ovarian cancer cell lines [10,17]. It was also found that there were higher levels of iNOS expression in less differentiated breast carcinomas [10,18]. The underlying mechanisms of the relationship between high iNOS expression and the pathogenesis of human breast cancer are not well defined. This review aims to address these mechanisms. We hypothesize that there are some cross-talk signaling pathways between iNOS expression and the expression of breast cancer protooncogenes: HER2, BRCA1, and BRCA2. In this report, we describe the molecular regulations of the expression of the breast cancer oncogenes HER2, BRCA1, and BRCA2 and the role of iNOS expression in those regulations. In addition, we performed bioinformatic analyses to delineate the interrelationships between iNOS and the expression of the oncogenes above.

## 2. Breast Cancer

Breast cancer is a heterogeneous disease that is the global leading cause of cancer-related death in women [19,20]. It is heterogeneous because it consists of many different subtypes that have different clinical outcomes [20,21,22]. As a result, there must be continuing research advancements in diagnosing and treating breast cancer [20,21,22]. Specifically, it is important to study tumor progression and resistance to treatments at the clinical and molecular levels [20,21,22]. Different subtypes of breast cancer include alterations in the gene expression of oncogenes such as HER2/neu, Ras, and PI3K [23].

Protooncogenes are the genes in normal cells that drive the cell cycle forward through cell proliferation and differentiation [24]. However, when protooncogenes undergo gain-of-function mutations, they become permanently activated, becoming oncogenes [24]. These oncogenes stimulate uncontrollable cell division, which promotes tumorigenesis in the development of cancer [23]. These protooncogenes that turn into oncogenes are HER2, BRCA1, and BRCA2.

### 2.1. Breast Cancer Protooncogenes

#### 2.1.1. HER2

There are many oncogenes involved in the development and progression of breast cancer. HER2 is an oncogene that is located on chromosome 17q and encodes the 185 kDa tyrosine kinase receptor protein [23,25]. HER2 is a protein that is a member of the epidermal growth factor receptor (EGFR) family [26,27]. The overexpression of HER2 is found in breast, ovarian, lung, and oral cancers [27,28,29,30,31,32,33,34]. Specifically, overexpression of HER2 has been found in 20%–30% of invasive human breast cancer cases [23,28,35].

##### Transcriptional Regulation of HER2

It has been shown that HER2 is overexpressed through the transactivation of its promoter [36,37]. One study found that E1A is capable of repressing the HER2*/neu* gene at the transcriptional level. E1A represses HER2 by inhibiting transcription factors that bind to and activate the promoter region of HER2 [37,38]. Specifically, E1A binds to p300/CBP and inactivates the p300/CBP complex on HER2 [37,39]. Since p300/CBP is an enhancer-binding protein for HER2, the inhibition of p300/CBP represses HER2 gene expression [37,40,41].

Another study investigated the effects of the SV40 large T-antigen (c-myc) on HER2 gene expression [42,43]. Hung et al. characterized the rat, mouse, and human HER2 promoters and used transient transfection assays [41,43,44,45,46,47]. Hung et al. found that when c-myc was transfected into the HER2 promoter regions, the rat HER2 promoter was inhibited while the human HER2 gene expression was unaffected [42,43,48,49]. As a result, Hung et al. concluded that there must be continuing large T gene therapy on HER2 in human cancer cells to study its regulation of the overexpression of HER2 [43].

Additionally, the effects of epigallocatechin-3 gallate (EGCG) on HER2/neu in breast cancer cells were investigated [50]. Pianetti et al. analyzed the focal mammary tumors of transgenic mice that overexpressed HER2/neu [50,51]. EGCG is a main antioxidant in green tea, and this study fed mice green tea to study the effects of EGCG on HER2/neu [50,52]. Through the use of the Akt kinase assay and immunoblot analysis, Pianetti et al. found that EGCG slows the proliferation and reduces the growth of tumor cells [50]. EGCG does this by reducing basal receptor tyrosine phosphorylation of HER2/neu [50]. EGCG also inhibits HER2/neu signaling pathways, including the pathway that activates NF-kB [50,53]. NF-kB causes inflammation in tumor cells, which contributes to the progression of cancer [50,53]. As a result, EGCG inhibits the growth and proliferation of mammary tumors by inhibiting HER2/neu signaling pathways [50]. It also methylates HER2/neu, which results in the inhibition of HER2/neu gene and protein expression [50].

##### Epigenetic Regulation of HER2

There are also epigenetic factors that affect the expression of HER2/neu. A study investigated how DNA methylation and demethylation affect HER2/neu expression in ovarian cancer [54,55]. The promoter region of HER2/neu has six CpG sites [55,56]. In this experiment, Hattori et al. methylated these CpG sites using specific primers in PCR that targeted positions 2, 206, 213, 299, and 513 in the HER2/neu promoter region [55,57]. Hattori et al. then used immunohistochemistry and found that methylating the HER2/neu promoter resulted in the silencing of the gene [55]. These results were then compared to the demethylated HER2/neu promoter, where it was found that demethylation increased HER2/neu gene expression [54,55]. This study was conducted by comparing the samples of 43 human ovarian cancers to 43 human non-cancerous ovarian tissues [55]. As a result, it was found that methylation of the promoter region of HER2/neu downregulates HER2 gene expression while demethylation upregulates HER2 gene expression [55].

##### Post-Transcriptional Regulation of HER2

There are also post-transcriptional factors that regulate HER2/neu gene expression [58]. Small interfering RNAs (siRNAs) are double-stranded RNAs that induce post-transcriptional silencing of specific targeted genes [58]. Yang et al. transfected u20bp miRNAs into HER2/neu of human breast cancer cells [58,59]. From the transfection, it was found that retrovirus-mediated RNA interference using siRNAs resulted in the gene silencing of HER2/neu [58]. As a result, siRNAs are able to decrease the expression of HER2/neu mRNA and protein, which can lead to inhibited tumor growth [58]. The siRNAs are synthesized to be homologous to regions of the HER2 exons [60]. Choudhury et al. also synthesized them to be homologous to other HER family members [60].

#### 2.1.2. BRCA1

##### Wild-Type BRCA1

BRCA1 functions as a tumor suppressor gene that controls cell cycle checkpoints and repairs DNA within a normal cell [61]. However, in breast and ovarian cancer cells, BRCA1 is mutated [61]. Normally, BRCA1 represses estrogen receptor α (ER-α), which is a transcription factor that can mediate tumorigenesis upon exposure to estrogen [61,62,63]. BRCA1 is a gene that has many important cellular functions within the body, including DNA repair, cell cycle regulation, and transcriptional regulation of other genes [64,65,66]. Specifically, it binds to complex DNA structures and regulates the G2/M checkpoint protein Chk1 [67,68,69]. It also can induce large-scale chromatin unfolding, which contributes to transcription and repair within the cell [68,70]. 

When BRCA1 is mutated, it no longer represses ER-α, which results in the development and progression of triple-negative breast cancer [61,62,63,71].

Additionally, BRCA1 normally functions to control the migration of breast cancer cells, which limits metastasis within the body [61]. The mechanism behind this is that BRCA1 ubiquitinates ezrin, radixin, and moesin (ERM), which is a membrane protein complex that promotes metastasis [61,72]. However, when BRCA1 is mutated in breast cancer cells, it can no longer degrade the ERM complex [61,73]. This results in the overexpression of ERM proteins, which leads to the metastasis of breast cancer cells from the prime site to the secondary site [61].

It was also found that in mammary epithelial cells, mutated BRCA1 promotes cell motility and invasion [61,74]. In these cells, BRCA1 enhances the protein expression of transcription factors Snail 1 and Snail 2, known as slug [61,75,76]. The mechanism behind BRCA1′s regulation of Snail 1 and Snail 2 remains unknown [75]. Upregulation of Snail 1 and Snail 2 elevates the level of epithelial-to-mesenchymal phenotype (EMT) in the cells [61,76]. EMT changes the shape of cells to be more spindle-shaped, making them highly motile [61]. As a result, the mutation of BRCA1 results in EMT being induced, which promotes metastasis of breast cancer cells [61]. Another study found that BRCA1 has a role in maintaining genetic stability in a normal cell [77].

Specifically, there are three domains of the BRCA1 protein that are mutated in many cancer cases [78,79]. One such domain is the RING domain that encompasses amino acids 1-109 (exons 2-7), which functions as an E3 ubiquitin ligase [78,79]. To test this, one study mutated the BRCA1 RING domain by replacing it with alanine in genetically engineered mice [79,80,81]. Clark et al. found that the E3 ubiquitin ligase activity and therefore the RING domain plays a large role in tumor suppression [79,80,81].

Another domain is the region encoded by exons 11-13, which encompasses over 65% of the BRCA1 sequence. It also encodes the nuclear localization sequences (NLS) and has binding sites for proteins such as the retinoblastoma protein (RB), c-Myc, Rad50, and Rad51 [79,82]. Researchers are continuing to investigate the exact structure and function of this domain, but Clark et al. have a general idea from assessing the functions of its binding partners [79,82]. One of its binding proteins includes Myc, which is a transcription factor for a large number of genes, where binding to this domain would activate other genes [79,82]. Additionally, Rad50, Rad51, and PALB2 are involved in DNA repair. Lastly, RB controls cell cycle progression [79,82]. As a result, the researchers concluded that the domain of exons 11-13 plays a large role in the tumor suppressor function of BRCA1 [79,82].

Finally, there is the BRCT domain from exons 16-24 (amino acids 1650-1863), which is a phosphoprotein-binding domain [79,83]. It has specificity for proteins that are phosphorylated by ATM/ATR kinases, which are both activated by DNA damage [79,83]. The phosphoproteins that bind to the domain are BACH1, CtIP, and CCDC98/abraxas [79,84,85,86]. The main function of the BRCT domain is to recognize the sequence pSer-X-X-Phe in its phosphorylated binding partners in order to modulate interactions between BRCA1 and phosphoproteins [79,84,85,86]. BRCT phosphorylates target phosphoproteins in response to DNA damage in the cell [79,84,85,86]. As a result, the mutation of this domain can lead to a loss of tumor suppressor function in BRCA1 [79].

However, when there is a deficiency of BRCA1, there is uncontrolled cell division, increased proliferation, and tumorigenesis [71,72,73]. Estradiol (E2) is an abundant estrogen that is found in women, and it was shown to induce high levels of BRCA1 during puberty and pregnancy in mice [87,88,89]. This is because E2 stimulation leads to the estrogen receptor (ER-α) and p300 binding to an activator protein site on the BRCA1 promoter [89,90]. As a result, estrogen regulates BRCA1 activity. However, it was also found that BRCA1 transcriptionally regulates ER-α, creating a negative feedback loop [89,91]. BRCA1 inhibits transcriptional activation of ER-α by deregulating p300, which is a coactivator of ER-α [89,91]. However, ER-α signaling pathways promote the proliferation and differentiation of breast and ovarian tissues [89]. When BRCA1 is mutated, most of the time it results in the inability of BRCA1 to repress ER-α [89,91]. As a result, tumorigenesis occurs, resulting in BRCA1-related malignancies in breast, ovary, and prostate tissues [66,89].

##### Mutated BRCA1

BRCA1 and BRCA2 mutations are known to contribute to the susceptibility of breast and ovarian cancers. Through genetic linkage studies, BRCA1 has been localized to chromosome 17q [92,93]. Specifically, BRCA1 accounts for 81% of breast–ovarian cancer families, while BRCA2 only accounts for 14% of these families [93,94,95]. However, studies have shown that the combination of BRCA1 and BRCA2 mutations accounts for the most high-risk breast cancer families [93,96]. This was found by using genetic markers to test for BRCA1 and BRCA2 mutations in families from the Breast Cancer Linkage Consortium (BCLC) [93].

When BRCA1 is mutated, it was found that it can indirectly promote tumorigenesis by increasing the mutation rates of other genes [70,97]. Xu et al. found that BRCA1 can induce mutations in the Trp53 tumor suppressor gene [70,98,99]. Specifically, the mutated BRCA1 inhibits the expression of Trp53 in mammary gland tissues [70,98,99]. As a result, the inhibition of Trp53 results in increased cell proliferation and therefore tumor formation in mammary cells [70,98,99]. Yasmeen et al. analyzed the effects of the 3450delCAAG BRCA1 mutation on breast carcinogenesis and metastasis [67]. Specifically, it was found that in human mammary epithelial cell lines, this mutation in BRCA1 promoted cell cycle progression, cell motility, and cell invasion [67]. Yasmeen et al. tested this by comparing the mutated cell lines to the normal BRCA1 cell lines using transfection, Western blotting techniques, and RT-PCR [67].

It was found that mutated BRCA1 deregulates cell cycle progression at the G0/G1 phase [67,100,101]. This is because BRCA1 deregulates cyclins A, E, D1, D2, and D3 along with their catalytic partners Cdk2, Cdk4, and Cdk6 [67,100,101]. As a result, mammary epithelial cells surpass the G0/G1 cell cycle checkpoint, allowing for uncontrolled cell cycle progression [67,100,101]. Additionally, mutated BRCA1 deregulates the expression of caveolin-1, P-cadherin, E-cadherin, and Id-1, which are major regulators of cell invasion and metastasis [67]. As a result, the 3450delCAAG BRCA1 mutation deregulates regulators of cell cycle progression, cell motility, and cell invasion, which can contribute to the metastasis of breast and ovarian cancers [67].

Another study found that mutated BRCA1 affects the expression of the β-subunit of human chorionic gonadotropin (β-hCG) in breast cancer [102,103,104]. β-hCG has been shown to inhibit apoptosis, promote cell invasion and proliferation, and act as an immunosuppressant [61,105,106,107]. When BRCA1 is mutated, it upregulates the expression of β-hCG. As a result, it allows for β-hCG to suppress immune responses through downregulating IL3, IL13R, TNF12, and TNF10 [61,108,109]. It also has been shown that the mutated BRCA1 induces β-hCG-mediated tumorigenesis through TGFBRII signaling [61]. Therefore, Sengodan et al. found that BRCA1 increases the expression of β-hCG, which is a molecule that induces metastasis of breast and ovarian cancer cells [61]. 

##### Transcriptional Regulation of Wild-Type BRCA1

There are also transcription factors that regulate the expression of BRCA1. The protein inhibitor of differentiation 4 (ID4) has been shown to downregulate the expression of BRCA1 in MCF-7 cells [68]. It was also found that the GA-binding protein (GABP)-α/β binds to the promoter and activates BRCA1 gene expression in MCF-7 cells and T47-D cells [68]. Additionally, the carboxyl-terminal binding protein 1 (CtBP1) has been found to be present in 92% of invasive breast cancer cases [110]. Through the use of nuclear staining of human breast tissue, transfections, and ChIP assays, Deng et al. found that CtBP1 downregulated BRCA1 at the transcriptional level [110]. It inhibits BRCA1 by binding to the promoter region, which enables CtBP1 to interact with DNA-binding proteins and co-repressor complexes such as CtIP [110]. As a result, BRCA1 mRNA levels are lower in cells with higher CtBP1 levels, which results in tumorigenesis [110].

There is also regulation of BRCA1 gene expression by the Rb-E2F pathway in murine and human cancers [111]. In BRCA1, there is a DNA-binding site for E2F directly upstream of exon 1, and when E2F binds to this site, it transcriptionally activates the BRCA1 pathway [111,112]. However, Rb—a product of the retinoblastoma susceptibility gene (RB1)—inhibits the expression of E2F by binding to and blocking the activation domain of E2F proteins [111,113,114,115]. Therefore, when Rb inhibits the expression of E2F, it can no longer activate BRCA1 transcription, resulting in decreased BRCA1 gene expression [111]. This inactivation of the tumor suppressor gene contributes to cancer development [111]. These results were found through the use of in vivo transfections of E2F1 in transgenic mouse models, Northern blot analyses, electrophoretic mobility shift assays (EMSA), and plasmid and luciferase reporter assays [111].

##### Epigenetic Regulation of Wild-Type BRCA1

There are also epigenetic factors that regulate the expression of BRCA1 [68]. In one study, the effects of methylation on the promoter region of BRCA1 were investigated [68,116]. The methylation of the promoter caused the promoter to be less accessible [68,117]. As a result, the inability of activators to bind to their respective sites on the promoter leads to decreased BRCA1 transcription and gene expression [68,116]. Rice et al. conducted this experiment using high-resolution bisulfite sequence analysis of 21 axillary node-negative breast cancer patient specimens [116].

Lu et al. used a candidate gene approach to study how hypermethylation affects BRCA1 gene expression [117]. It was found that BRCA1 has a CpG island in its 5′ region of the promoter [117]. When this CpG island is hypermethylated, it results in the silencing of the expression of the BRCA1 gene [117,118,119].

The effects of hypoxia on BRCA1 gene expression because hypoxia often occurs in tumor microenvironments were investigated [120]. Specifically, Lu et al. examined the histone modifications that were affected by hypoxia [120]. Using ChIP assays and reporter constructs, Lu et al. analyzed the cells MCF-7, A549, RKO, and HCC 38 [120]. Lu et al. found that hypoxia increased H3K9 methylation and decreased H3K9 acetylation at the promoter region of BRCA1 [120]. Therefore, hypoxia can regulate BRCA1 expression by transcriptionally repressing the promoter through histone methylation and acetylation [120].

Bosviel et al. found that the metabolite S-equol had an effect on the gene expression of BRCA1 and BRCA2 in MCF-7 and MDA-MB-231 cells [121]. This resulted in the transcriptional activation of BRCA1 and BRCA2, increasing gene expression and tumor suppressor function [121]. As a result, demethylation activates gene expression in BRCA1 and BRCA2 [121].

##### Post-Transcriptional Regulation of BRCA1

There are miRNAs that regulate BRCA1 expression in triple-negative sporadic breast cancer cases [122]. Specifically, miR-146a and miR-146b-5p are two miRNAs that bind to the same site in the BRCA1 promoter region of the mRNA [122]. Using reporter assays and transfection of miRNAs in mammary cell lines, it was found that miR-146a and miR-146b-5p both downregulate the expression of BRCA1 [122]. Garcia et al. concluded this because they found that the miRNAs increased proliferation and decreased homologous recombination, which are two impaired processes that are normally regulated by BRCA1 [122].

Other post-transcriptional factors include the miR-15/107 group of miRNAs, which regulates the BRCA1 coding sequence in primates and rodents [123,124]. In this study, the miR-15/107 group of miRNAs were transfected into cell lines HT-29 and MCF-7 [124]. After using transient transfection, Quann et al. used quantitative reverse transcriptase PCR, luciferase validation of miRNA targets, Western immunoblotting, and statistical analyses to analyze the effects of the miRNAs on BRCA1 gene expression [124]. Eight of ten miRNAs in the miR-15/107 group downregulate BRCA1 mRNA abundance [124,125]. The miRNAs repress BRCA1 by degrading its RNA targets and repressing the translation of messenger RNAs (mRNAs) [124,126,127]. As a result, the miR-15/107 group of miRNAs is a repressor of BRCA1 gene expression through its coding sequence [124]. 

#### 2.1.3. BRCA2

BRCA2 is a large protein made of 27 exons and 3418 acids that is localized to the nucleus in MCF7 cells [128,129,130]. There have been studies that have shown that BRCA2 may play a role in the regulation of transcription [129,131]. There has also been a study that has shown that BRCA2 is involved in DNA repair and recombination by binding to rad51 [129,132]. Bertwistle et al. found that BRCA2 is cell cycle-regulated and is induced at the late G1/early S phase, which is before DNA synthesis [129,133]. However, since BRCA2 is also involved in DNA repair, this means that during DNA synthesis its role could solely be to maintain genome integrity during replication [129,133]. Hence, BRCA2 is a tumor suppressor gene like BRCA1 [129].

##### Transcriptional Regulation of BRCA2

Mutations of BRCA2 have been linked to tumorigenesis in murine sporadic breast cancers [134,135]. In the murine BRCA2 gene, there is a region 148 bp upstream of the first exon that is necessary to activate transcription of BRCA2 in mammary cells [135,136]. It was also found that there is a 52 bp fragment between regions −92 and −40 bp that is necessary for promoter activity [136]. This is because it contains a CREB/ATF-binding site, where the CREB transcription factor family can bind to this region in the promoter to activate gene transcription [136]. Specifically, the transcription factors CREB-1, ATF-1, and CREM bind to their binding site on BRCA2 to activate gene expression [136]. Callens et al. inhibited these transcription factors, where a decrease in BRCA2 gene expression was observed [136]. Therefore, the CREB family upregulates BRCA2 gene expression [136].

There are DNA-damaging agents that regulate BRCA2 promoter activity in breast cancer cell lines [137]. Specifically, Wu et al. focused on the effects of adriamycin (ADR) and mitomycin C (MMC) on BRCA2 promoter activity [137]. Wu et al. found that ADR downregulates BRCA2 in a p53-dependent manner [137]. This means that the presence of ADR and MMC results in p53 inhibiting the USF transcription factor from binding to the BRCA2 minimal promoter, resulting in the downregulation of BRCA2 promoter activity [137]. This downregulation decreases BRCA2 mRNA and protein levels in the cell as a result of DNA damage induced by agents such as MMC and ADR [137].

##### Epigenetic Regulation of BRCA2

There are also epigenetic factors that regulate BRCA1 and BRCA2, such as hypermethylation [138]. Lobanova et al. used molecular genetic studies of 50 breast cancer tissues, each in different stages [138]. In 34% of the breast cancer cases, the promoter region of BRCA1 was hypermethylated [138]. The BRCA2 promoter region was hypermethylated in 50% of their cases [138]. In this case, hypermethylation inhibited BRCA1 and BRCA2 expression by blocking transcription factors from binding to the promoter [117,118,119]. Therefore, the promoter cannot be activated and mRNA and protein levels decrease [138]. This inhibits the function of BRCA1 and BRCA2 as tumor suppressor genes that regulate the cell cycle [130]. As a result, hypermethylation of BRCA1 and BRCA2 promoters can lead to the tumorigenesis of cells [138].

Dworkin et al. identified that methylation of BRCA2 can also affect BRCA2 gene expression [139]. In this study, 15 tumors that lacked the loss of heterozygosity (LOH) of the BRCA2 wild-type allele were analyzed [139]. Through sampling these tissues and analyzing their mutation type, it was found that silencing of BRCA2 through methylation was not very common in tumor cells [139]. However, in the tumor cells where BRCA2 was silenced, methylation silenced BRCA2 by binding to the promoter region and inhibiting other transcription factors from binding, like hypermethylation [139]. 

##### Post Transcriptional Regulation of BRCA2

There are also post-transcriptional factors that regulate BRCA2 expression through interactions with miRNAs [140]. Specifically, one study by Mogilyansky et al. tested the interactions of the miR-17/92 cluster with the mRNA of BRCA2 in pancreatic, breast, colon, and kidney tissue cell lines [140]. Luciferase reporter assays were used to find that in the cluster, miR-19a and miR-19b interact with the 3′UTR region of BRCA2′s mRNA [140]. The overexpression of these miRNAs resulted in a decrease in BRCA2 mRNA levels and therefore a decrease in BRCA2 protein levels in these cell lines [140]. Therefore, miR-19a and miR-19b both downregulate BRCA2 gene expression by directly decreasing mRNAs [140].

There are also lncRNAs that are involved in regulating the repairment of double-stranded DNA breaks, which can promote tumorigenesis [141]. One known lncRNA is PCAT-1, which is a cytoplasmic lncRNA that is induced by genotoxic stress [141,142,143]. Presner et al. found that PCAT-1 regulates the expression of BRCA2 in prostate cancer cells [141]. PCAT-1 post-transcriptionally represses the BRCA2 3′UTR region of the mRNA, which disrupts homologous recombination (HR) [142].

## 3. iNOS/NO

### 3.1. General

**Figure 1:**
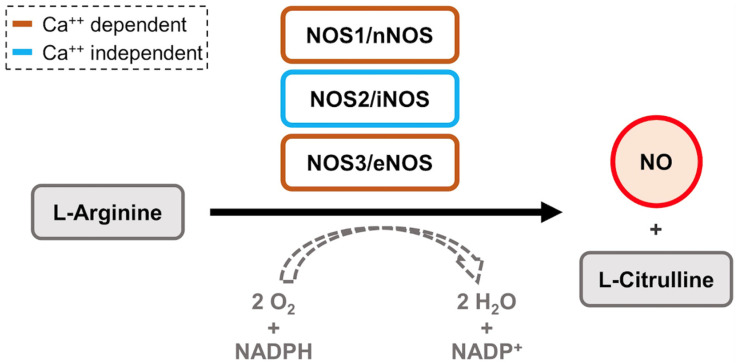
NOS enzymatic reaction. The chemical reaction catabolized by all the three human NOS enzymes involves the formation of L-citrulline and NO from L-arginine and in presence of O_2_ and NADPH. The three known human NOS genes are: NOS1 or neuronal NOS (nNOS), NOS2 or inducible NOS (iNOS), and NOS3 or endothelial NOS (eNOS).

Inducible nitric oxide synthase (iNOS, NOS2) is an enzyme that catalyzes the production of large amounts of nitric oxide (NO) by L-arginine [144,145]. Specifically, it oxidizes L-arginine to produce L-citrulline and NO [146,147] (Figure 1). L-Arginine uptake and availability are controlled by the cationic amino acid transporters CAT1, CAT2, and CAT3 [148]. These transporters control L-arginine uptake, which in turn regulates the production of NO by iNOS [148]. iNOS is a main source of NO in the body and regulates the immune system [149,150].

Unlike eNOS and nNOS, iNOS is not regulated by calcium levels in the body [150,151,152] (Figure 1). In order to activate eNOS and nNOS expression, calcium (Ca2+) has to bind to calmodulin (CaM) to allow CaM to bind to the CaM-binding domains of eNOS and nNOS [153,154]. However, in iNOS, CaM is naturally tightly bound to the CaM-binding domain without the need for Ca2+ [147,155]. As a result, iNOS is unregulated by intracellular Ca2+ levels and therefore is a major contributor to the overproduction of NO in the body [140,150]. The positive effects of NO overproduction include defending the host against viral or microbial pathogens. In the Vaccinia virus, NO inhibits DNA synthesis by inhibiting the activity of DNA-synthesizing enzymes such as ribonucleotide reductase (RR) [156,157]. NO inhibits the activity of RR by scavenging the tyrosyl radical that is necessary for the catalysis of RR [158]. As a result, there are decreased levels of Vaccinia protein synthesis. This was found by generating the Vaccinia virus recombinant that expressed murine iNOS in vitro [157,159,160]. The negative effects include contributing to the pathogenesis of inflammatory diseases such as atherosclerosis [149,150,161,162,163]. iNOS is expressed in atherosclerotic lesions, where NO reacts with superoxide anion, causing oxidative damage that leads to cellular damage and inflammation [162,164,165].

There are different functions of NO synthesized by iNOS, where the concentration level of NO can either protect against disease or contribute to the pathogenesis of disease [166,167,168]. iNOS producing NO can have immunoregulatory effects by functioning as an intra- and intercellular signaling molecule that can inhibit or enhance the immune response [149,160,169]. NO binds to and induces a conformational change in the enzymes involved in immune responses. The conformational change can either activate the enzyme or hinder the ability of the enzyme to catalyze the reaction [149]. However, the expression of iNOS is also involved in the pathogenesis of immune diseases. This is seen in the L. donovani infection in the liver, where the expression of iNOS inhibits T cell proliferation or induces T cell apoptosis, furthering the progression of infection [149,169,170,171].

#### 3.1.1. Transcriptional Regulation of iNOS 

The human *iNOS* gene is located on chromosome 17 and consists of 26 exons and 25 introns [172,173]. *iNOS* gene expression is regulated at different levels, including transcriptional regulation [173,174]. A number of transcription factors were reported to bind the iNOS gene promoter, resulting in either the activation or the inhibition of *iNOS* transcription [169,175].

In Table 1, one of the first transcription factors identified as a direct transcriptional inducer of the *iNOS* gene is the nuclear factor (NF)-κB [169,176]. In addition, iNOS transcription was found to be under the regulation of the STAT-1α pathway. When IFN-γ is present in the cell, it activates cytoplasmic JAKS that tyrosine phosphorylates the transcription factor STAT-1α. This process enables STAT-1α to dimerize, translocate to the nucleus, and activate iNOS gene expression [169,177,178,179].

The cAMP-induced transcription factors bind to the C/EBP-binding sites at positions −155 to −163 bp on the murine iNOS promoter [180]. In addition, AP-1 is a transcription factor that regulates murine iNOS expression by binding to the iNOS promoter at position −1125 bp. However, in human iNOS, overexpression of AP-1 was shown to inhibit iNOS promoter activity using a supershift assay [169,175].

The octamer factor binds to the iNOS promoter region 24 bp upstream from the TATA box and activates iNOS transcription in murine and rat cells [169,181,182,183]. The peroxisome proliferator-activated receptors (PPARs) inhibit iNOS promoter activity indirectly by inhibition of NF-kB or AP-1 through competition for CBP/p300 in human chondrocytes and murine macrophage cells [169,184,185,186].

The tumor suppressor p53 inhibits iNOS promoter activity through transcriptional trans-repression of the promoter in human and murine epithelial cells [169,187,188]. The hypoxia-induced factor-1 (HIF-1) enhances iNOS promoter activity by binding to the HIF-1 site on the promoter in murine macrophages [169,189,190]. The retinoic acid receptor-α (RAR-α) uses RXR-PPAR-γ heterodimers to inhibit murine iNOS activity [169,191,192,193]. The estrogen receptor-β (ER-β) mediates the transcriptional activation of the murine iNOS promoter, which was found using an immunoblot analysis [169,193,194]. 

Long non-coding RNAs (lncRNAs) were further validated to control iNOS gene expression. Specifically, the effects of the intergenic lncRNA named Nostrill on iNOS gene expression were analyzed in human microglia [195]. When Nostrill expression is induced by cytokine LPS [195,196,197,198], it drives secondary and late iNOS gene transcription [195,199,200]. It can also scaffold the RNA polymerase II at the iNOS promoter region, enhancing the efficiency of the promoter, ultimately increasing iNOS transcription [195,201,202,203].

#### 3.1.2. Epigenetic Regulation

The human iNOS gene is also regulated at an epigenetic level. It was found that DNA methylation played a large role in the transcriptional silencing of the iNOS gene at its promoter [204,205].

Acetylation is another epigenetic factor that regulates iNOS gene expression [206]. Hypermethylation of the iNOS gene promoter can regulate iNOS gene expression [207]. Demethylation is another epigenetic factor that can regulate the expression of the iNOS gene in human articular cartilage cells [208]. To study the effects of demethylation, one study used transfection of human articular cartilage samples and fluorescence-activated sorting [208] (Table 1).

#### 3.1.3. Post-Transcriptional Regulation

iNOS expression is further regulated at a post-transcriptional level mainly through destabilization of its transcript [209]. Furthermore, several microRNAs have been reported to negatively regulate iNOS gene expression, directly or indirectly through facilitating mRNA degradation or inhibiting translational activity and protein synthesis (Table 1).

#### 3.1.4. iNOS-Derived NO Levels in Cells

NO is a signaling molecule that is produced by NO synthases eNOS, nNOS, and iNOS [146,147]. Specifically, iNOS is responsible for the regulation of NO in the immune system [149,150]. iNOS transcription is induced by cytokines such as LPS and IFN-γ [210,211]. One experiment used the direct measurement of NO release using an NO-specific amperometric probe and a cyclic AMP assay [211]. They found that low NO basal levels are always present in the cytoplasm of the cell [210,211,212]. It also takes 3–4 h for NO levels to be increased by iNOS induction [210,211,212]. They conducted a 48 h experiment, where they found that iNOS is capable of producing and maintaining higher NO levels for 24–48 h after induction before returning to basal levels [210].

After NO is produced by iNOS, it has a half-life in the range of 9–900 min [213,214]. This half-life is a result of physiological NO reacting with oxygen (O2) to produce nitrite (NO2-) [215]. Nitrite is then further oxidized to form nitrate (NO3-), where NO is metabolized [215]. This results in the return of NO levels in immune cells to return to basal levels [215]. In biological systems, the metabolic rate of NO to nitrite and nitrate is dependent on both oxygen concentrations and ambient NO concentrations [215]. As a result, the shortest half-life of NO occurs after iNOS is induced in immune cells, where NO concentrations are at the highest levels [210,214].

### 3.2. iNOS-Induced NO Effects

The induction of iNOS increases levels of exogenous NO, which can lead to the S-nitrosylation of different transcription factors. S-nitrosylation is the process of covalently attaching a nitric oxide moiety to a cysteine thiol. This process can result in regulating the function and expression of different proteins [216,217].

Reynaert et al. investigated the effects of iNOS-induced NO S-nitrosylation of the NF-kB family of transcription factors [217,218]. NF-kB transcription factors play a large role in immune and inflammatory responses [217]. It also regulates cell survival and proliferation [174]. S-nitrosylation by NO in NF-kB inhibits NF-kB-dependent gene transcription, promoter activity, and DNA binding [217,219,220].

### 3.3. iNOS/NO Functions in Cancers

iNOS-induced NO plays many roles in cancer development [16]. One study found that iNOS-induced NO upregulates the expression of matrix metalloproteinase 2 (MMP2), MMP-0, and VEGF, which promote metastasis [16].

Kielbik et al. found that iNOS-induced NO can also lead to the progression of ovarian cancer [4,221,222]. NO suppresses BRCA1 and BRCA2 promoter activity, which decreases their mRNA expression [4]. BRCA2 is another tumor suppressor gene that is involved in DNA repair [129].

Saed et al. analyzed epithelial ovarian carcinoma (EOC) cells in their pro-oxidant state [219,220,221,222,223,224,225,226]. In this state, there is increased expression of both iNOS and NO, and when iNOS was induced by L-arginine, it resulted in lower apoptosis in the EOC cells [223,224,225,226]. The mechanism behind this was that iNOS-induced NO S-nitrosylated caspase-3. Caspase-3 is a lysosomal enzyme that is involved in apoptosis for the cells, where S-nitrosylation decreases its activity and therefore decreases apoptosis in EOC cells, which results in the progression of ovarian cancer [223,224,225,226].

Sha and Marshall investigated how iNOS-derived NO-dependent S-nitrosylation post-transcriptionally modified proteins [227]. The effects on the p53 tumor suppressor protein were studied [227]. p53 is modified by Hdm2-mediated proteasomal degradation [227,228]. However, when NO is present, it S-nitrosylates Hdm2, inhibiting the Hdm2 pathway [227,229]. Therefore, it increases p53 levels in the cell through S-nitrosylation of Hdm2, which is a ubiquitin ligase [227,229]. It has also been found that S-nitrosylation of the protein S100B can increase p53 activity because S100B is a binding partner of p53 [227,228].

Jia et al. investigated how iNOS-induced NO can S-nitrosylate GAPDH, which is a key glycolytic enzyme [230,231]. When GAPDH is S-nitrosylated, it regulates enzyme activity [230,231]. iNOS induces the S-nitrosylation of GAPDH at Cys152 or Cys247 in response to the activation of the S100A8/A9 complex [231]. Additionally, it was found that the S-nitrosylation of GAPDH dysregulates the IFN-γ translational pathway, which is a pathway that exhibits anti-tumor properties [231,232]. As a result, iNOS-induced NO S-nitrosylation can regulate the expression of target proteins that can result in inflammation and cytotoxicity within different cells [231].

## 4. iNOS and Breast Cancer Implications

### 4.1. Triple-Negative Breast Cancer (TNBC)

**Figure 2:**
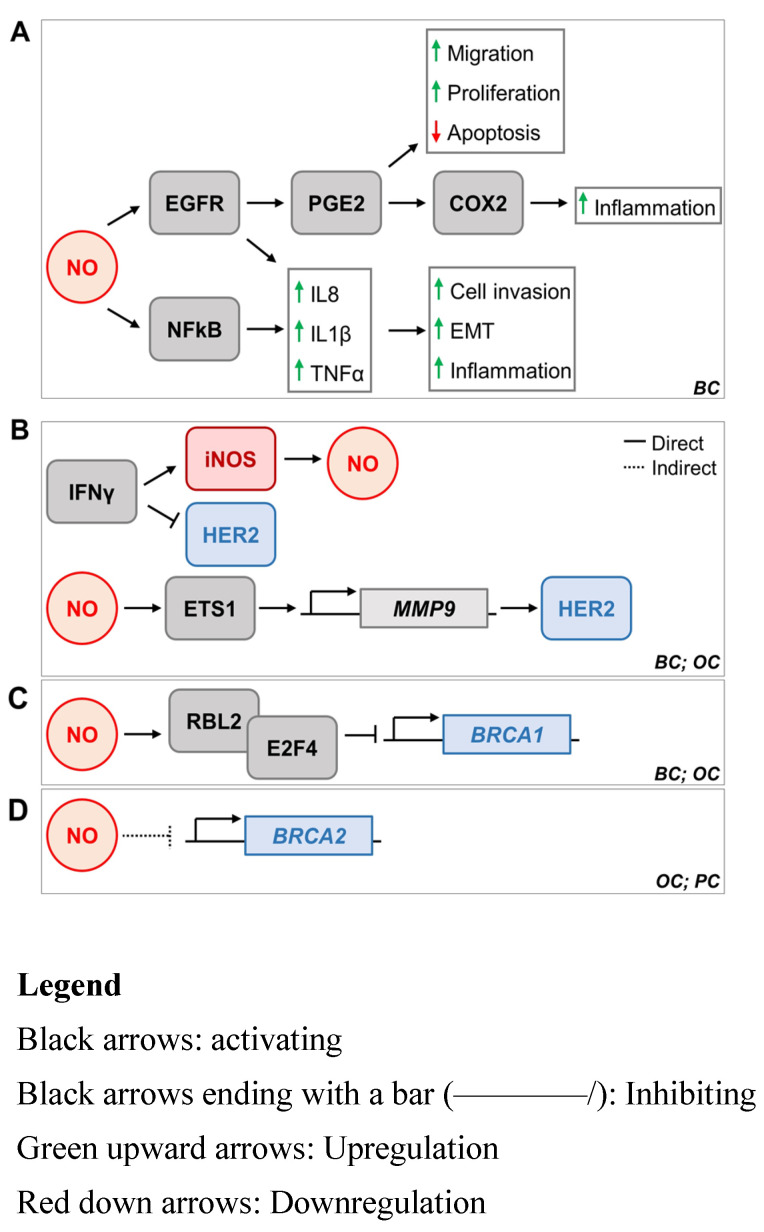
Role of NO in breast cancer (BC) and other relevant cancers. (**A**) Pathways regulated by NO in BC and relative intracellular NO-mediated effects. (**B**) Direct (plain lines) and indirect (dotted lines) pathways correlating NO with HER2 (top), (**C**) BRCA1 (middle), and (**D**) BRCA2 (bottom). OC, ovarian cancer; PC, pancreatic cancer; EMT, epithelial–mesenchymal transition.

iNOS-induced NO has been shown to contribute to the progression of basal-like triple-negative breast cancer (TNBC) [233,234]. NO induced mutations in p53 and activated the epidermal growth factor receptor (EGFR) through S-nitrosylation [234,235,236,237]. Specifically, this S-nitrosylation resulted in the phosphorylation and therefore activation of the EGFR/ERK/MAPK pathway [14,236]. When this pathway is phosphorylated, it activates the metabolite prostaglandin E2 (PGE2) [14,238,239]. PGE2 was found to promote cell migration, invasion, and proliferation on top of inhibiting apoptosis [14,238,239]. PGE2 also leads to COX-2 overexpression, which promotes inflammation in cells [233,236]. This is important because inflammation is a major driver of cancer metastasis [240]. These results are schematically represented in Figure 2.

In TNBC, Garrido et al. found that iNOS-induced NO activated the NF-kB pathway and increased the secretion of cytokines IL-8, IL-1β, and TNFα [14,241,242,243]. Interestingly, the activation of the EGFR pathway induced the expression of these cytokines [14]. IL-8 has been shown to cause cell invasion, metastasis, and epithelial-to-mesenchymal transition [14,236,244]. IL-1β and TNFα have cytotoxic effects in the cell and promote tumor progression [14,245,246,247,248]. NF-kB promotes inflammation in cancer cells [50,53]. Overall, iNOS actives the EGFR/ERK/MAPK pathway, which results in the activation of PGE2, COX-2, IL-8, IL-1β, TNFα, and NF-kB [14]. All of these factors promote the progression of cancer through metastasis, cell invasion, and inflammation [14]. 

iNOS expression regulates the expression of TNBC biomarkers [15]. Chen et al. used shRNA-guided knockdown to downregulate iNOS in TNBC cells [15]. When iNOS was knocked down, tumor marker CD1 along with special TNBC biomarkers RUNX1 and BCL11A were downregulated [15]. The iNOS knockdown also upregulated the tumor suppressor CK1 [15]. As a result, the knockdown of iNOS can partially reverse the tumorigenesis of TNBC cells [15].

Chen et al. found similar effects when iNOS was inhibited by NG-monomethyl-L-arginine monoacetate (L-NMMA) and 1400 W [15,249]. The inhibition of iNOS decreased cell proliferation, cell migration, and cancer stem cell self-renewal [15,249]. iNOS overexpression can also result in colon adenoma, enhanced KRAS-induced lung carcinogenesis, inflammation, tumor growth, and glioma stem cell proliferation [15,188,250,251].

### 4.2. ER Breast Cancer

In estrogen receptor-negative (ER-) breast cancer, it was found that Ets-1 is a transcriptional mediator of NO signaling [16,243]. Ets-1 promotes tumorigenesis by activating the Ras/MEK/ERK signaling pathway [16,237]. Ets-1 is a proto-oncogene that promotes angiogenesis and extracellular matrix remodeling [16,252,253,254]. It is activated through binding to the MMP-9 gene [16,255]. MMP-9 expression also promotes HER2 oncogenic expression [16,256,257]. As a result, when Ets-1 is activated, it binds to and activates MMP-9 expression, which in turn also activates HER2 [16,255,256,257]. Therefore, there is an indirect relationship between NO and HER2 as a result of them both being mediated by the transcription factor Ets-1 [16]. These results are shown above in Figure 2B.

Mishra et al. analyzed the effects of eNOS-induced NO on the progression of cancer [16]. In the presence of estrogen, eNOS activates the phosphatidylinositol 3-kinase (PI3K)/Akt/eNOS signaling pathway [16,258,259]. It also activates the ERK-1/2 pathway [16,258,259]. Both of these pathways have signaling-mediated effects that promote breast cancer [16,258,259]. It was also found that in the breast cancer subtype invasive ductal carcinoma, NO biosynthesis was upregulated in higher-grade tumors [16,17].

## 5. iNOS and Breast Cancer Oncogenes HER2, BRCA1, and BRCA2

iNOS-derived NO has been found to contribute to the progression of breast cancer [12,13]. Specifically, iNOS-derived NO can disrupt DNA repair mechanisms and can cause genomic instability [13,260,261,262,263]. It has been found that the rise in NO levels can alter the levels of cell proliferation and apoptosis in cells [13,234,264]. This can lead to mutations that are linked to the initiation, promotion, and progression of cancer [13].

### 5.1. iNOS and HER2

The relationship between IFN-γ, iNOS, and HER2 expression was investigated by Marth et al. [228]. IFN-γ induces the expression of iNOS [265]. However, IFN-γ decreases the expression of the HER2 oncogene [265,266]. This was against the predictions of the study because iNOS is often related to the induction of oncogenes [265]. Marth et al. suspected that this lack of correlation could be due to the fact that iNOS is dependent on the activation of other signaling pathways besides IFN-γ [265,267,268]. Therefore, other signaling pathways could be necessary to establish the relationship between iNOS and HER2 [265,267,268].

### 5.2. iNOS and BRCA1

Yakovlev et al. analyzed how iNOS affects the function of BRCA1 in breast cancer cells [13,269]. BRCA1 is a tumor suppressor gene that is involved in cell cycle regulation and DNA repair [71,72,73]. The presence of iNOS-induced NO led to the dephosphorylation of RBL2 in the promoter region of BRCA1 [13,270,271]. This causes the formation of the RBL2/E2F4 complex in the same region [13,270,271]. As a result, E2F4 replaces E2F1 in binding to the BRCA1 promoter [13,270,271]. This is important because E2F1 is an activator while E2F4 is an inhibitor, where this shift causes the inhibition of BRCA1 promoter activity [13,270,271]. These results are shown above in Figure 2C.

Therefore, BRCA1 cannot perform homologous recombination repair, resulting in the increase in error-prone nonhomologous end joining (NHEJ) in the cell [13]. The increase in mutations causes inflammation, carcinogenesis, and genomic instability, which all contribute to the progression of breast cancer [13]. Yakovlev et al. came to these conclusions by performing cell cultures with MCF-10A, AF49, and RAW264.7 cells [13].

Plenchette et al. found that iNOS-induced NO can alter BRCA1 tumor suppressor activity through NO donors [13,272]. Specifically, the NO donors S-nitroso-N-acetylpenicillamine (SNAP) and diethylenetriamine NONOate (DETANONOate) have been found to promote the inhibition of BRCA1 expression [13,272]. This results in the hindrance of the ability of BRCA1 to repair DNA through the HR pathway, which leads to NHEJ and tumorigenesis [13,272].

### 5.3. iNOS and BRCA2

Garcea et al. found that iNOS-induced NO contributed to the inhibition of 8-OH-dg adducts [1,273]. This inhibition results in the promotion of pancreatic cancer cell growth [1,273]. However, BRCA1 and BRCA2 contribute to the repair of these same 8-OH-dg adducts [1,274]. Therefore, iNOS-induced NO indirectly suppresses BRCA1 and BRCA2 tumor suppressor functions [1,274]. Kielbik et al. also found that iNOS directly inhibits the promoter activity of BRCA1 and BRCA2 in ovarian cancer cells. As a result, this decreases the expression and function of BRCA1 and BRCA2 [4].

The relationship between iNOS and BRCA2 was found in ovarian and pancreatic cancer cell lines. However, little is known about the relationship between iNOS and BRCA2 in breast cancer cell lines. Therefore, further research is necessary to investigate whether or not the relationship between iNOS and BRCA2 in breast cancer cell lines follows the same trends as that of pancreatic and ovarian cancer cell lines. These results are shown above in Figure 2D.

## 6. Implication of iNOS, HER2, and BRCA1/2 in CSC Pathophysiology

Cancer stem cells (CSCs) constitute a small fraction of cancer cells within the tumor bulk that possesses pluripotent and renewing properties [275]. CSCs are thought to be responsible for driving oncogenesis, disease progression, metastasis, and therapeutic resistance [275]. Recent findings have confirmed the contribution of NO metabolism in defining the “stemness” properties of CSCs through cross-regulation of “stemness”-associated signaling pathways, including the Notch and Wnt cascades [276]. Therefore, molecules able to modify the maintenance of a stem cell phenotype, including NO, are of great research and therapeutic interest.

Although initial reports have demonstrated that iNOS-generated NO is a distinctive feature of CSCs originated mainly by tumors developed in an inflammatory background, further studies demonstrate differential expression and function of NOS isoforms in CSCs that mainly depend on tumor type and aggressiveness. For example, in hepatocellular carcinoma, iNOS (NOS2) overexpression in cancer cell lines and human tissues promotes NOTCH-1-mediated stemness and tumor initiation in vivo, through a cGMP/PKG dependent mechanism [277]. Treatment of lung cancer cells with NO concentrations ranging between 20 and 40 μM, which are similar to those produced by iNOS, was able to induce dedifferentiation of lung cancer cells towards a stem-cell-like phenotype through stabilization of critical CSC-associated markers, such as Oct4 [278]. Accordingly, selective inhibition of the high endogenous iNOS expression in TNBC cells significantly reduced CSC self-renewal ability, tumor initiation, and the number of lung metastases as a result of EMT inhibition [279]. Furthermore, targeting of endogenous NO production by iNOS silencing in ER+ breast cancer cells displayed inhibition of mammosphere formation and expression of CSC-associated markers, while it significantly reversed tumor resistance to tamoxifen treatment [280].

**Figure 3:**
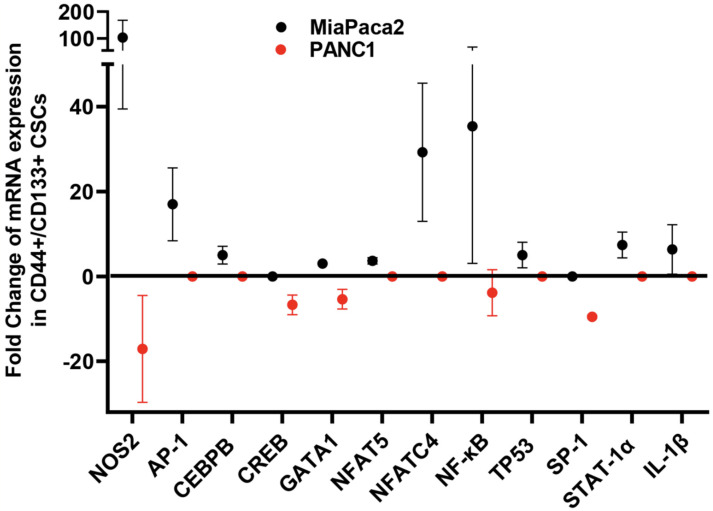
mRNA differential expression of iNOS and iNOS-inducing genes in CD133+/CD44+ CSCs isolated by the PDAC cell lines MiaPaca2 and PANC1. For gene microarray analysis, Agilent Array platform was employed. Quantile normalization and subsequent raw data processing were performed using the GeneSpring GX v11.5.1 software package (Agilent Technologies). Differentially expressed mRNAs between compared samples (CD44+/CD133+ vs. CD44-/CD133-) of each cell line were identified through fold change (FC) filtering (FC ≥ 2 was set as a cut-off value). Columns represent mean fold change of gene expression ± SDEV.

Our preliminary findings obtained by gene microarray analysis of a CD133+/CD44+ CSC population isolated from the pancreatic adenocarcinoma (PDAC) cell lines PANC1 and MiaPACA2 revealed a strong iNOS mRNA induction in MiaPaca2-derived CSCs compared to non-SC population (CD133-/CD44-), whereas in PANC1 CSCs, iNOS was significantly inhibited (Figure 3). Furthermore, iNOS overexpression in MiaPaca2 CSCs was positively correlated with a concurrent significant increase in the mRNA expression of reported co- and trans-activators of iNOS transcription, including AP-1, CEBPB, GATA 1, NFAT5, NFATC4, NF-κΒ, STAT-1a, TP53, and IL-1β (Figure 3). Respectively, iNOS downregulation in PANC1 CSCs was associated with a reduction in the mRNA levels of GATA 1, NF-κΒ, Sp1, and CREB (Figure 3). The observed difference in the iNOS expression profiles between the two cell lines may be attributed to the diversity of tumor aggressiveness, with MiaPaca2 depending more on high iNOS levels in order to sustain the aggressive phenotype [281].

In contrast, colon cancer mesenchymal cells do not express iNOS and instead overexpress eNOS that impairs the CSC phenotype and induces tumor cell proliferation [282]. The above findings indicate that the type and expression levels of each NOS isoform in CSCs might be specific to cancer type and tumor aggressive potential, as well as the impact of inflammation-induced iNOS and NO in tumorigenesis. In addition, more precise studies on purified CSC populations are needed for drawing safer conclusions on iNOS impact on cancer “stemness”.

Downregulation of BRCA1 has been associated with a significant increase in the CSC-like populations in breast cancer cells, whereas BRCA1 reconstitution increases cell sensitivity to HDAC inhibitor-induced loss of stemness, thus suggesting that BRCA1 functions as a breast stem cell regulator, while its status may determine tumor response to therapy [283]. Likewise, the key role of altered (overexpressed/amplified) HER2 signaling in the maintenance and enrichment of breast CSCs, through crosstalk with stemness-related pathways, has been highlighted in several reports [284]. A splice variant of full-length HER2 mRNA and a collection of HER2 truncated carboxy-terminal fragments, known as d16HER2 and p95HER2, respectively, have been characterized as the most oncogenic HER2 isoforms with significant implications in the regulation of HER2^+^ breast CSC features, including tumor initiation, EMT induction, and resistance to targeted therapy [284]. However, no direct associations between iNOS, BRCA1, and HER2 expression profiles have been established so far in any type of CSCs, including breast CSCs.

**Figure 4:**
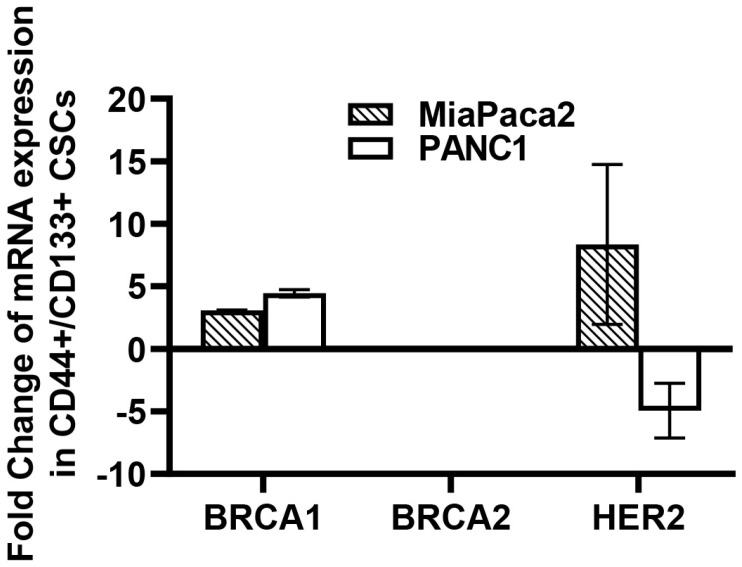
Differential expression of BRCA1, BRCA2, and HER2 mRNA transcripts in CD133+/CD44+ CSCs isolated by the PDAC cell lines MiaPaca2 and PANC1. For gene microarray analysis, Agilent Array platform was employed. Quantile normalization and subsequent raw data processing were performed using the GeneSpring GX v11.5.1 software package (Agilent Technologies). Differentially expressed mRNAs between compared samples (CD44+/CD133+ vs. CD44-/CD133-) of each cell line were identified through fold change (FC) filtering (FC ≥ 2 was set as a cut-off value). Columns represent mean fold change of gene expression ± SDEV.

Given the direct implication of BRCA1/2 signaling in the pathophysiology of hereditary pancreatic adenocarcinoma (PDAC) [285], as well as the recently reported prognostic impact of HER2 expression or amplification in the survival of PDAC patients [286], we examined possible alterations in the expression profiles of BRCA1/2 and HER2 mRNAs in our PDAC CSC models. Both MiaPaca2 and PANC1 cell lines are proficient in BRACA1/2 wild-type expression [287], while HER2 levels are more profound in MiaPaca2 than PANC1 [288]. CD133+/CD44+ CSCs from both cell lines significantly overexpress BRCA1 compared to the corresponding CD133-/CD44- non-SC populations, whereas HER2 overexpression was observed only in MiaPaca2-derived CSCs. In contrast, PANC-1-derived CSCs showed significant inhibition of HER2 mRNA expression (Figure 4). No significant differences in BRCA2 mRNA expression were detected in our CD133+/CD44+ enriched CSC populations from both cell lines. Overall, our findings from our PDAC CSC model suggest a positive correlation of iNOS, BRCA1, and HER2 expression in CSCs of aggressive tumors that may be critical for sustaining cell “stemness” and associated properties (Figure 4).

## 7. Bioinformatic Analyses: Correlation between BRCA1/2 Mutations and Genes Involved in the NOS Pathway 

Through the development of high-throughput technologies for the analysis of molecular alterations associated with tumor development, a huge amount of bioinformatics data has been generated and collected in publicly available databases [289]. Among these, The Cancer Genome Atlas (TCGA) consortium collects clinical information as well as gene expression, ncRNA expression, DNA methylation, and protein expression data of 33 different human cancers [290].

The analyses of these data have allowed the identification of novel potential diagnostic and prognostic biomarkers for different tumors through the identification of specific genes, miRNAs, or proteins dysregulated in cancer [291,292,293]. 

Despite the availability of all these molecular data also for breast cancer, only a few studies have filtered patients according to the presence of *BRCA1/2* mutations or the amplification/overexpression of ErbB2. Similarly, no in-depth correlation studies have been performed between the expression levels of amplified or mutated genes such as ErbB2 and BRCA and the expression levels of genes involved in the NOS pathway.

To establish the correlation existing between genetic alterations affecting *BRCA1*, *BRCA2* and ErbB2 with the expression levels of NOS-associated genes, the phenotype and gene expression data contained in the TCGA Breast Cancer (TCGA BRCA) database were evaluated. In particular, the TCGA BRCA database contains 24 different datasets collecting clinical and molecular information on a total of 1247 breast cancer patients [294].

The datasets “Phenotypes” and “IlluminaHiSeq Gene Expression RNAseq” were downloaded in order to identify breast cancer patients with mutations affecting *BRCA1* or *BRCA2* or amplification affecting ErbB2 and the expression data of 20,530 different genes, respectively. 

**Figure 5:**
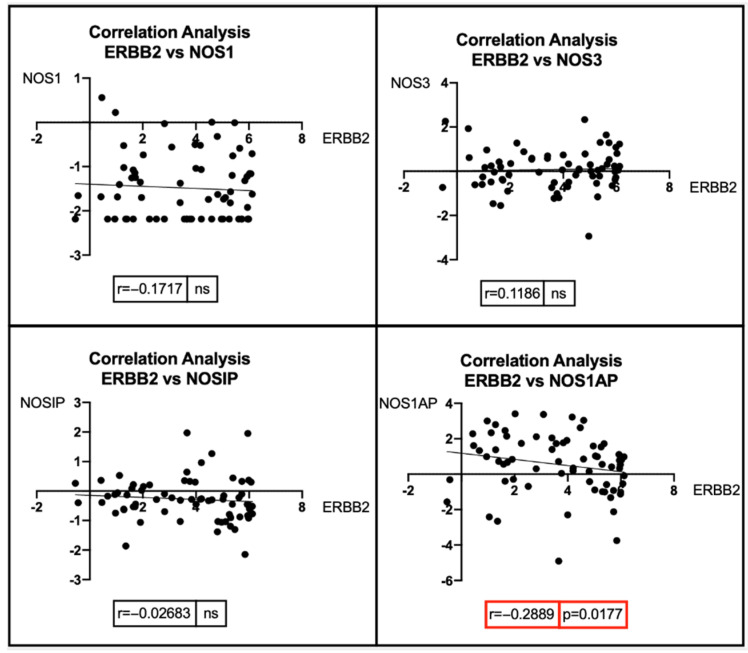
Correlation analyses between the expression levels of ErbB2 and NOS genes in TCGA breast cancer patients with HER2 amplification. Each black dot represents a cancer patient.

Through the “Phenotypes” dataset, 25 *BRCA1* and 22 *BRCA2* mutated patients were identified. Similarly, a total of 68 breast cancer patients with ErbB2 amplification were identified, of which 14 had a HER2 FISH ratio > 2.2 and 47 had a HER immunohistochemistry (IHC) score of 3+ or higher. Seven patients had both a HER2 FISH ratio > 2.2 and a 3+ IHC score. For these subsets of patients, the expression levels of *BRCA1*, *BRCA2*, and *ErbB2* were obtained by analyzing the “IlluminaHiSeq Gene Expression RNAseq” dataset. From the same dataset, the expression levels involved in the NOS pathway were observed, including NOS1, NOS3, nitric oxide synthase interacting protein (NOSIP), and nitric oxide synthase 1 adaptor protein (NOS1AP). Of note, NOSIP negatively regulates the production of nitric oxide by inducing NOS1 and NOS3 translocation to the actin cytoskeleton, thus inhibiting their enzymatic activity [295], while NOS1AP is mainly involved in the modulation of neuronal NO through the regulation of *NOS1* with other proteins [296]. Unfortunately, no expression data about *NOS2* were recorded on the “IlluminaHiSeq Gene Expression RNAseq” dataset; therefore, this gene was not investigated (Figure 5).

**Figure 6:**
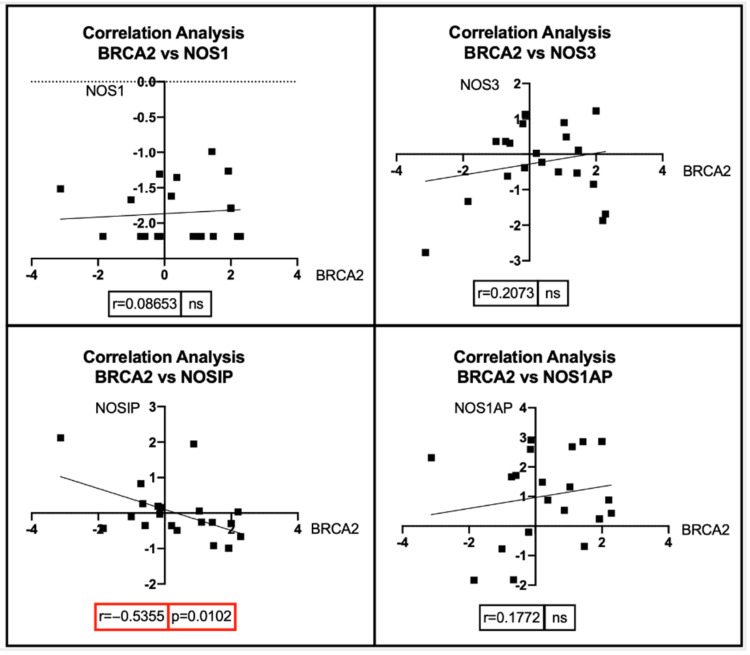
Correlation analyses between the expression levels of BRCA2 and NOS genes in TCGA breast cancer patients with mutations affecting BRCA2. Each black square represents a cancer patient.

Pearson’s correlation and Spearman’s correlation analyses were performed for the three groups of breast cancer patients, i.e., BRCA1-mutated, BRCA2-mutated, and ErbB2-amplified breast cancer patients, depending on the normal or non-normal distribution of expression data (Figure 6).

**Figure 7:**
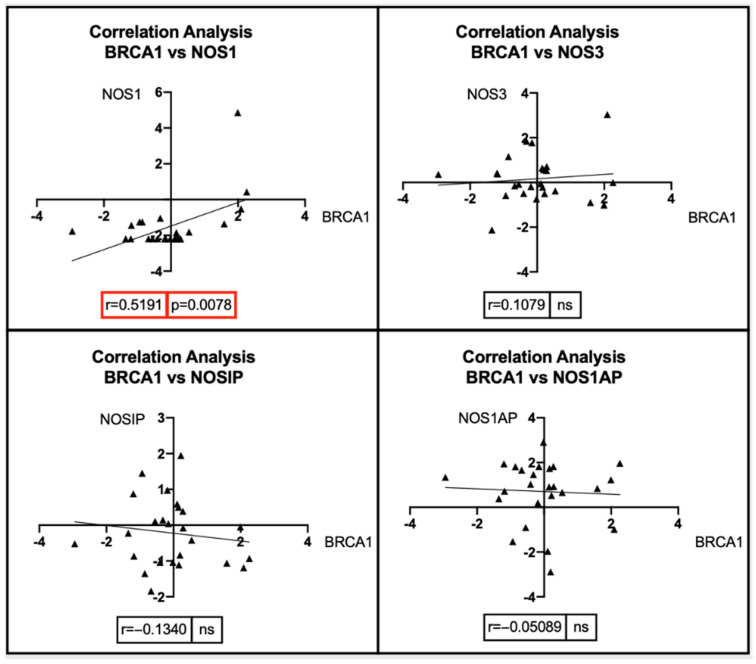
Correlation analyses between the expression levels of *BRCA1* and *NOS* genes in TCGA breast cancer patients with mutations affecting *BRCA1*. Each black triangle represents a cancer patient.

The correlation analyses between the expression levels of *BRCA1* and NOS genes revealed how the expression levels of dysregulated *BRCA1* due to somatic mutations positively correlate with the expression levels of NOS1 (r = 0.5191; *p* = 0.0078), while no significant correlations were observed between *BRCA1* and *NOS3*, *NOSIP*, and *NOS1AP* (Figure 7).

## 8. Discussion and Perspectives

It has been demonstrated that human breast cancer tissues express high levels of iNOS. This expression has predicted increased tumor progression and a poor outcome of survival in women with estrogen receptor alpha-negative (ER-negative) tumors [193,297]. More recently, the same group of investigators reported that the co-expression of iNOS and COX2 enhances tumor growth and shortens the survival of patients with ER-negative breast cancer [298].

Several mechanisms have been suggested for the role of iNOS as a driver of breast cancer progression. For instance, high iNOS expression has been correlated with high p53 mutations. Additionally, iNOS-derived NO activates several survival signaling pathways, promotes HIF1-alpha stabilization (tumor cells cope with hypoxia), and mediates immunosuppression and metastasis [298]. In TNBC, iNOS-induced NO resulted in mutations of p53 and activation of EGFR. This was through S-nitrosylation leading to the activation of the EGFR/ERK/MAPK and NF-kB pathways [16]. These activated pathways led to tumor cell proliferation, migration, invasion, and resistance to cytotoxic drugs [16].

Interestingly, the relationship between iNOS expression and the expression of breast cancer protooncogenes HER2, BRCA1, and BRCA2 in the pathogenesis of breast cancer is not well understood. Herein, we have discussed this relationship based on reported literature. In addition, we have also used bioinformatics to examine the possible linkage between the NOS pathway and the expression of the protooncogenes.

In ER-negative breast cancer, the protooncogene ETS-1 is a transcriptional mediator of NO and promotes angiogenesis. Activated Ets-1 binds and activates MMP9 expression, which in turn activates and increases the expression of HER2. Thus, a linkage is observed between NO and HER2. However, in other reports, it was found that while IFN-g induces the expression of iNOS, it also inhibits the expression of HER2 [298]. It is possible that depending on the stimulus, various signaling pathways may result in either the activation or inhibition of HER2 expression. It will be useful to ascertain the various conditions under which NO activates or inhibits HER2 expression in order to develop appropriate interventions that regulate iNOS expression.

Regarding the relationship between iNOS/NO and BRCA1, it was found that NO indirectly inhibits BRCA1 promoter activity, and NO can alter BRCA1 tumor suppressor activity. Similar findings were reported that NO suppresses the tumor suppressor activity of BRCA2 [13]. Clearly, iNOS-mediated NO affects the tumor suppressor activities of both BRCA1 and BRCA2 as well as potentiating the expression of HER2. These effects result in the promotion of tumor growth and malignancy. While many stimuli from the tumor microenvironment increase iNOS expression in normal cells, it is possible that such an increase is involved in the initial trigger of oncogenesis, alone or with other factors.

Several mechanisms have implicated the roles of NO and RNS in the induction of tumorigenesis. For instance, NO leads to oxidative nitrosative stress that promotes DNA damage, suppression of DNA-repair enzymes, post-translational modification of proteins, and the formation of nitrosamines [11,299]. In addition, mutations in various genes have been reported to be strong genetic risk factors for ovarian cancer progression [4]. Interestingly, BRCA1 and BRCA2 mutations are responsible for the development of about 90% of all ovarian cancers [222]. Genes controlling cell growth, DNA repair processes, and apoptosis (e.g., TP53, BRCA1, BRCA2, and PARP) appear to be prime targets for mutations by NO/RNS.

Additionally, the expression of iNOS in breast CSCs is associated with malignancy and tumor growth. Its selective inhibition has been shown to reduce CSC self-renewal capacity [257]. In a recent study, Lopez-Sanchez et al. have investigated the resistance of ER-positive breast cancer CSCs to anti-hormonal therapy (tamoxifen) [280]. Lopez-Sanchez et al. examined the role of NO in CSC characteristics and examined whether targeting NO in breast CSCs would have any effect on sensitivity to tamoxifen. Silencing of NO or iNOS in CSCs resulted in the inhibition of mammospheres and CSC biomarker expression and sensitized the CSCs to tamoxifen-mediated cytotoxicity. These findings were consistent with the findings that tamoxifen-resistant cells exhibited overexpression of iNOS and NOTCH-1 when compared to parental cells.

Kaplan–Meier survival analysis confirmed these in vitro findings. This analysis found that low NOS2 expression was associated with lowered metastasis in ER+ breast cancer patients that were treated with tamoxifen. Clearly, both tumor type and iNOS expression levels in CSCs dictate the aggressiveness and malignancy of cancer cells. Thus, targeting iNOS is a therapeutic strategy to treat breast cancer, alone or in combination with cytotoxic drugs [300]. Of interest, the expression of BRCA1 in breast cancer inversely correlates with the frequency of the CSCs [301]. In contrast, overexpression of HER2 correlates with enrichment of CSCs [284]. It will be useful to investigate the associations of iNOS, BRCA1, and HER2 expression in CSCs.

Similar findings to those observed in breast cancer were also found in bladder cancer CSCs [302]. In bladder cancer CSCs, high expression of iNOS was associated with increased invasion and tumor recurrence. Additionally, inhibition of iNOS inhibited tumor progression and reduced the number of CSCs. Therefore, the findings demonstrated that iNOS plays a central role in bladder cancer progression and the maintenance of CSCs.

Bioinformatic analyses did not reveal the correlations between iNOS, mutated BRCA1/2, and overexpression of HER2 in breast cancer datasets. However, the expression levels of gene products in the NOS pathway were able to be detected, namely NOS1, NOS3, NOSIP, and NOS1AP. The expression levels of mutated BRCA1 correlated with the expression of NOS1 with no significant correlations with NOS3, NOSIP, and NOS1AP.

Noteworthily, several studies have already demonstrated the pivotal role of NOS1 in the development and progression of different tumors. Indeed, its overexpression is associated with ovarian cell proliferation and invasion as well as with chemoresistance [303,304]. Further studies confirmed the association between NOS1 expression and drug resistance in other tumors such as melanoma where NOS1 overexpression led to a low response to interferon [305,306]. As regards breast cancer, no studies on the effect of neuronal NOS (nNOS/NOS1) are reported in the literature; however, it was demonstrated how NOS1AP is able to bind other proteins, SCRIB and VANGL1, regulating different features of breast cancer cells including cell polarity, migration, and progression suggesting how this protein could be involved in the pathogenesis of this tumor when dysregulated [307]. In addition, other studies on neurodegenerative disorders demonstrated the strict interaction existing between NOS1AP and NOS1 identifying potential targetable binding sites useful to regulate the effect of the interactions of these two proteins [308]. Similarly, also the interactions existing between NOS1 and NOSIP have been investigated; however, the functional effects of these interactions in cancer are not fully understood yet [309,310]. All these data together with the most recent findings on the efficacy of nitric oxide-targeted therapy in estrogen receptor-positive breast cancer cells [280] suggest that the entire NO pathway could play a key role in breast cancer.

Clearly, further studies must be done in order to examine the relationship between the various subsets of breast cancer and iNOS. While it is clear that the overexpression of iNOS is associated with breast cancer and other cancer malignancies, there are also reports that the expression of iNOS/NO is also associated with tumor suppression. Vannini et al. have reviewed the dual role of NO in cancer and reported several cancer models where the expression of NO inhibited tumor growth and metastasis [3]. In addition, the iNOS expression by non-cancer cells in the TME was associated with tumor inhibition and reduced metastases in model systems.

## 9. Conclusions

Overall, the above findings have indicated that iNOS/NO is involved in the regulation of the protooncogenes, HER2, BRCA1, and BRCA2. NO and RNS also play a role in promoting gene mutations. Therefore, clearly both BRCA1 and BRCA2 are susceptible to mutations and play a large role in the pathogenesis of breast and ovarian cancer. More investigations are warranted to examine the direct relationship between iNOS/NO, BRCA1/2 mutations, and the onset of tumor development. Likewise, the roles of iNOS/NO in the regulation of HER2 transcription, expression, and onset of tumorigenesis are to be determined. Such investigations may be useful for therapeutic interventions at early stages and also as preventive measures in the future.

## Figures and Tables

**Table 1:** Regulation of iNOS.

Transcriptional Factors: Name	Type of Factor	Expression
NF-kB	Pre-transcriptional factor	Upregulation
STAT-1α	Pre-transcriptional factor	Upregulation
cAMP-induced transcription factors	Pre-transcriptional factors	Upregulation
AP-1	Pre-transcriptional factors	Downregulation in human iNOSUpregulation in murine iNOS
Octamer factor	Pre-transcriptional factors	Upregulation
PPAR	Pre-transcriptional factors	Downregulation
p53	Pre-transcriptional factors	Downregulation
HIF-1	Pre-transcriptional factors	Upregulation
RAR-α	Pre-transcriptional factors	Downregulation
ER-β	Pre-transcriptional factors	Upregulation
Nostrill (lncRNA)	Transcription factor	Upregulation
DNA methylation	Epigenetic post-transcriptional regulation	Downregulation
Histone H3K9 methylation	Epigenetic post-transcriptional regulation	Downregulation
Hypermethylation	Epigenetic post-transcriptional regulation	Downregulation
Demethylation	Epigenetic post-transcriptional regulation	Upregulation

## Abbreviations

8-OH-dg	Deoxyguanosine
A549	Adenocarcinomic human alveolar basal epithelial cells
ADR	Adriamycin
Akt	Automated turbidimetric-kinetic
AP-1	Activating protein-1
ATF 2	Activating transcription factor 2
ATM	Ataxia-telangiectasia mutated
ATR	ATM and Rad-3 related
β-hCG	Beta human chorionic gonadotropin
BACH1	BTB domain and CNC homology 1
BCL11A	B-cell leukemia/lymphoma
BCLC	Breast Cancer Linkage Consortium
BRCA1/2	Breast cancer susceptibility gene
Bp	Base pair
BRCT	BRCA1 C terminus
Ca2+	Calcium
CaM	Calmodomin
CAT	Chloramphenicol acetyltransferase
CBP	CREB binding protein
CCDC98	Coiled-coil domain-containing protein
CD1	Cluster of differentiation 1
Cdk 2/4/6	Cyclin-dependent kinase 2/4/6
CEBPBs	CCAAT-enhancer-binding proteins
cGMP/PKG	Cyclic guanosine monophosphate/protein kinase G
ChIP	Chromatin immunoprecipitation
Chk1	Checkpoint protein 1
CK1	Casein kinase 1
C-myc	Cellular myc
cNOS	Constitutive nitric oxide synthase
COX-2	Cyclooxygenase 2
CREB	cAMP-response element binding protein
CREM	cAMP-responsive element modulator
CSC	Cancer stem cell
CtBP1	Carboxyl-terminal binding protein 1
CtIP	COUP-TF interacting protein
Cys	Cysteine
DNA	Deoxyribonucleic acid
E1A	Adenovirus early region 1A
E2/E3	Estradiol 2/3
E2F1/4	E2 transcription factor 1/4
EGCG	Epigallocatechin-3
EGFR	Epidermal growth factor receptor
EMSA	Electrophoretic mobility shift assay
eNOS/NOS3	Endothelial nitric oxide synthase
EOC	Epithelial ovarian carcinoma
ER	Estrogen receptor-negative breast cancer
ERα	Estrogen receptor
ER-β	Estrogen receptor-β
ErbB2	Erythroblastic oncogene B2
ERK	Extracellular-signal-regulated kinase
ERM	Ezrin, radixin, and moesin
EMT	Epithelial–mesenchymal transition
Ets-1	Erythroblast transformation specific-1
FISH	Fluorescence in situ hybridization
G2/M	Growth 2/mitosis
GABP-α/β	GA binding protein
GAPDH	Glyceraldehyde-3-phosphate-dehydrogenase
GATA-1	GATA-binding factor-1/erythroid transcription factor
H3K9	Histone H3 lysine 9
HCC 38	Hepatocellular carcinoma
HDAC	Histone deacetylases
Hdm2	Human double minute 2
HER2/neu	Human epidermal growth factor receptor 2
HR	Homologous recombination
HT-29	Human colon adenocarcinoma cell line
ID ¼	Inhibitor of differentiation 1/4
IFN-γ	Interferon gamma
IHC	Immunohistochemistry
IL-1/2/4/8	Interleukin 1/2/4/8
IL-1B	Interleukin 1 beta
iNOS	Inducible nitric oxide synthase
KRAS	Kristen rat sarcoma viral oncogene homolog
L. donovani	Leishmania donovani
lncRNA	Long non-coding RNA
L-NMMA	NG-monomethyl-L-arginine monoacetate
LOH	Loss of heterozygosity
MAPK	Mitogen-activated protein kinase
MCF-7	Michigan Cancer Foundation-7
MEK	Mitogen-activated extracellular signal-regulated kinase
miRNA	MicroRNA
MMC	Mitomycin C
MMP 0/2/9	Matrix metalloproteinase 0/2/9
mRNA	Messenger RNA
ncRNA	non-coding RNA
NF-kB	Nuclear Factor kappa-light-chain-enhancer of activated B cells
NFAT-5/C4	Nuclear factor of activated T cells 5/C4
NHEJ	Nonhomologous end-joining
NLS	Nuclear localization sequences
nNOS/NOS1	Neuronal nitric oxide synthase
NO	Nitric oxide
NOSIP	Nitric oxide synthase interacting protein
NOS1AP	Nitric oxide synthase 1 adaptor protein
NOTCH-1	NOTCH homolog 1
Oct	Octamer factor
PANC-1	Human pancreatic cancer cell line
p53	Tumor suppressor p53
PALB2	Partner and localizer of BRCA2
PARP	Poly(ADP-ribose) polymerase
PCAT-1	Prostate cancer associated transcript 1
PCR	Polymerase chain reaction
PDAC	Pancreatic adenocarcinoma
PGE2	Prostaglandin E2
PI3K	Phosphoinositide 3 kinase
qPCR	Quantitative polymerase chain reaction
Ras	Rat Sarcoma Virus
RB	Retinoblastoma protein
Rb-E2F	Retinoblastoma protein family-activator E2F
RBL2	RB Transcriptional corepressor like 2
RING	Really interesting new gene
RKO	Rectal carcinoma cell line
RNA	Ribonucleic acid
RNAseq	RNA sequence
RNS	Reactive nitrogen species
RR	Ribonucleotide reductase
RT-PCR	Reverse transcription PCR
RUNX1	Runt-related transcription factor 1
S phase	Synthesis phase
S100B	S100 calcium-binding protein B
shRNA	Short hairpin RNA
siRNA	Small interfering RNA
Slug	Snail 1/2
Sp-1	Specificity protein 1
SNAP	S-nitroso-N-acetylpenicillamine
STAT-1α	Signal transducer and activator of transcription 1
SV40	Simian vacuolating virus 40
TCGA	The Cancer Genome Atlas
TGFBRII	Transforming growth factor beta receptor type II gene
TME	Tumor microenvironment
TNBC	Triple-negative breast cancer
TNF	Tumor necrosis factor
TNF-α	Tumor necrosis factor alpha
Trp53	Transformation related protein 53
TUNEL	Terminal deoxynucleotidyl transferase dUTP nick end labeling
USF	Upstream stimulatory factor
UTR	Untranslated region
VEGF	Vascular endothelial growth factor
